# Using Large-Scale Sensor Data to Test Factors Predictive of Perseverance in Home Movement Rehabilitation: Optimal Challenge and Steady Engagement

**DOI:** 10.3389/fneur.2022.896298

**Published:** 2022-06-20

**Authors:** Edgar De Jesus Ramos Muñoz, Veronica Ann Swanson, Christopher Johnson, Raeda K. Anderson, Amanda R. Rabinowitz, Daniel K. Zondervan, George H. Collier, David J. Reinkensmeyer

**Affiliations:** ^1^Department of Mechanical and Aerospace Engineering, Henry Samueli School of Engineering, University of California, Irvine, Irvine, CA, United States; ^2^Department of Biomedical Engineering, Henry Samueli School of Engineering, University of California, Irvine, Irvine, CA, United States; ^3^Shepherd Center, Virginia C. Crawford Research Institute, Atlanta, GA, United States; ^4^Department of Sociology, Georgia State University, Atlanta, GA, United States; ^5^Moss Rehabilitation Research Institute, Philadelphia, PA, United States; ^6^Flint Rehabilitation Devices, LLC, Irvine, CA, United States; ^7^Department of Anatomy and Neurobiology, UC Irvine School of Medicine, University of California, Irvine, Irvine, CA, United States

**Keywords:** rehabilitation, stroke, exercise, telemedicine, therapy, engagement, perseverance

## Abstract

Persevering with home rehabilitation exercise is a struggle for millions of people in the US each year. A key factor that may influence motivation to engage with rehabilitation exercise is the challenge level of the assigned exercises, but this hypothesis is currently supported only by subjective, self-report. Here, we studied the relationship between challenge level and perseverance using long-term, self-determined exercise patterns of a large number of individuals (*N* = 2,581) engaging in home rehabilitation with a sensor-based exercise system without formal supervision. FitMi is comprised of two puck-like sensors and a library of 40 gamified exercises for the hands, arms, trunk, and legs that are designed for people recovering from a stroke. We found that individuals showed the greatest perseverance with the system over a 2-month period if they had (1) a moderate level of motor impairment and (2) high but not perfect success during the 1st week at completing the exercise game. Further, a steady usage pattern (vs. accelerating or decelerating use) was associated with more overall exercise, and declines in exercise amount over time were associated with exponentially declining session initiation probability rather than decreasing amounts of exercise once a session was initiated. These findings confirm that an optimized challenge level and regular initiation of exercise sessions predict achievement of a greater amount of overall rehabilitation exercise in a group of users of commercial home rehabilitation technology and suggest how home rehabilitation programs and exercise technologies can be optimized to promote perseverance.

## Introduction

The World Health Organization estimated that one in three individuals worldwide have conditions that would benefit from rehabilitation (1). Movement-related conditions, such as low back pain (~568 M people per year) and stroke (~80 M people per year) account for over 80% of these conditions (1). For stroke patients in the US, the total estimated cost for rehabilitation services is >$9B each year (2).

In the current rehabilitation service paradigm, clinicians instruct patients to continue practicing selected movement exercises on their own at home following periods of inpatient and/or outpatient treatment. Clinicians usually provide patients with printed descriptions of the exercises. The importance of continuing with therapeutic exercise at home has increased because of decades-long actions aimed at reducing inpatient rehabilitation stays (3). However, there have been few innovations that have helped ensure that discharged patients complete home rehabilitation exercise programs. The COVID-19 pandemic caused an even greater emphasis on carrying out rehabilitation at home (4–6), potentially furthering this trend toward expecting rehabilitation to occur outside of formal facilities in the longer-term (7).

Studies examining home exercise programs have found that compliance is partial across a variety of health conditions. While estimates vary, most reports indicate the majority of patients do not fully adhere to prescribed home exercise routines. One estimate suggested that up to 65% of patients are non-adherent to their home exercise programs (8). Therapist estimates of their patients' adherence tend to be lower than patients' reports. For example, only 36% of physical therapists reported high levels of adherence to home exercises (9). In a study of home exercise for low back pain, 39% of patients reported adherence, while therapists estimated 16% were adherent. Low adherence is also implied by patients' poor memory of their prescribed exercises: only 15% of participants were able to recall all of the exercises contained within their program and demonstrate them accurately (10).

Most home rehabilitation adherence studies have relied on subjective and self-reported methods, primarily surveys (8, 11). The introduction of sensor and computer gaming technologies for home rehabilitation—or mRehab (mobile rehabilitation) systems (12, 13)—has made it possible to objectively quantify adherence. Studies with sensor systems have reinforced the concept that adherence is partial and highly variable. A recent systematic review of home-based, upper limb practice after stroke examined 42 studies that used a variety of technologies to facilitate movement practice, ranging from the Wii, to the iPad, to custom-designed sensor or robotic devices (14). These studies were typically small: only three enrolled more than 30 participants, and the largest study, which used Nintendo Wii Sports for arm rehabilitation after stroke, enrolled 235 individuals (15). The studies also varied substantially on whether and how they prescribed a dose of practice. The seven studies that allowed participants to self-select their dose of practice found that stroke survivors chose to train for approximately 24 min/day, 4–5 days/week. For studies where practice amounts were prescribed, participants were asked to complete between 9.5 and 161 h of practice over four to 24 weeks. Adherence varied widely, being ≤ 50% in five studies, 51 to 74% in nine studies, 75 to 100% in 13 studies, ≥ 101% in six. Considering this variability, determining how to help patient populations consistently persevere with home exercise is an important goal for promoting health and function.

A key factor that has been hypothesized to influence the motivation to engage with rehabilitation exercise is the challenge presented by the assigned exercises (16). For example, if an individual is severely impaired, exercises can quickly become overly challenging, requiring large amounts of effort to complete (17, 18). On the other hand, exercises that are too easy to complete may be viewed as non-beneficial by the person exercising. In a study of repetitive finger movement training after stroke in which the exercise was gamified, success at playing the game predicted the level of self-reported motivation for engaging in the exercise, as well as self-efficacy in achieving functional gains (19).

**Figure 1:**
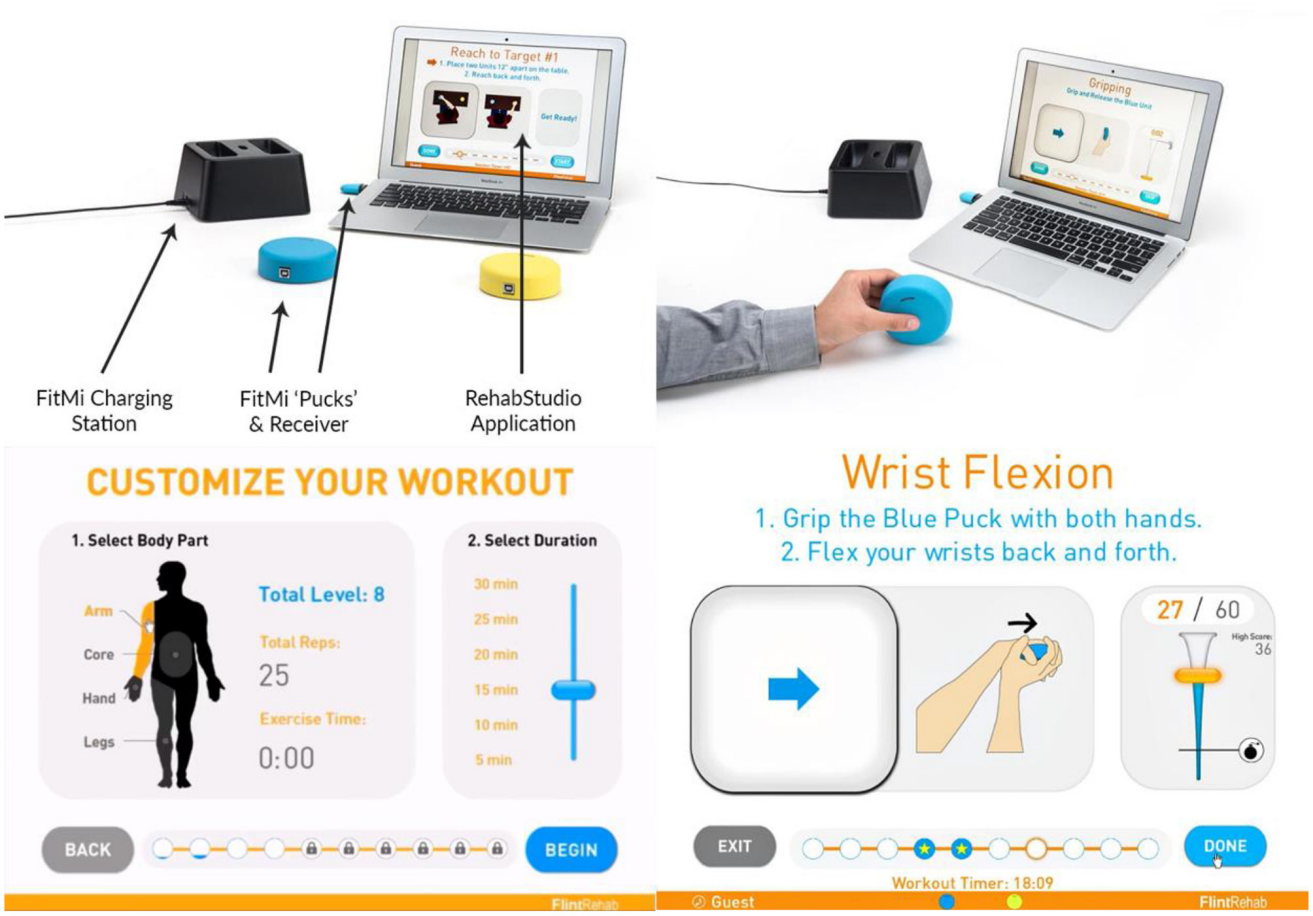
FitMi (produced by the company Flint Rehab Devices) consists of two force and motion sensing pucks and a software application called RehabStudio. Top row: Hardware required for FitMi, Bottom row: Graphical user interface for FitMi.

Here, we leveraged a unique opportunity to analyze anonymous usage logs from a commercial, sensorized, home rehabilitation technology, called FitMi, to study what predicts perseverance in rehabilitation. FitMi is comprised of two puck-like sensors and software that visually guides the user through 40 therapeutic exercises for the hands, arms, legs, and torso in a game-like setting (Figure 1). This game format allows users to “level-up” if they perform a target number of repetitions for a given exercise, providing a quantitative measure of successful exercise completion. Users typically buy the system out-of-pocket and use it freely on their own without direct supervision from a rehabilitation therapist. We are therefore studying a group of people who have taken concrete steps to continue their rehabilitation by acquiring a home rehabilitation technology. Individuals with enhanced autonomy and self-efficacy have better health outcomes, including in stroke rehabilitation (20–23). However, we hypothesize that even among this motivated subpopulation, there will be variance in their perseverance which is influenced by challenge level and steadiness of use.

To test whether challenge levels are associated with perseverance in unsupervised, home rehabilitation exercise, we studied whether the impairment level of the user, measured with the device itself, as well as the user's success in leveling up in the 1st week of use, predicted total amount of use of the system. Therapists sometimes warn patients not to “overdo” their exercises when they begin a new program lest they become too fatigued or sore. Further, people who engage with new consumer technologies are known to sometimes experience a novelty effect in which engagement is initially high but rapidly tapers (24, 25). We therefore also quantified steadiness of FitMi use and tested for a potential association with perseverance.

## Methods

We analyzed usage data from 2,581 users of the FitMi movement rehabilitation system acquired over a 3-year period. We required users to have had the system for at least 8 weeks to be included in the analysis. Data were anonymous, having been automatically uploaded to a server managed by Flint Rehabilitation Devices, the company that manufactures and sells FitMi, without any identifying information after each exercise session. The study was confirmed by the UC Irvine Institutional Review Board.

### FitMi Overview

FitMi is an FDA-listed medical device marketed to persons who have experienced a stroke to help them perform movement exercise. FitMi consists of two 8.9 cm diameter “pucks” that sense movement (using a 3-axis accelerometer and a 3-axis gyroscope) and compression force (using a load cell). Users hold the pucks or place them on a table or the floor in various configurations to exercise (Figure 1). The companion software uses data from the pucks' sensor arrays to detect completion of the exercises available in the system.

FitMi provides user-selectable, therapist-designed exercises for the arms, hands, core, and legs. Each of these four regions has ten possible exercises. Users initiate a session by selecting a body region and an amount of time to exercise: 5, 10, 15, 20, 25, or 30 min. FitMi then presents the unlocked exercises for that body area and the user chooses which exercises to perform during the session. If they achieve the target number of repetitions for a specific exercise in a set amount of time, they “level up” and the software will increase the target number of repetitions for the exercise. Once a user reaches level 10 (the maximum level) in an exercise, they go into “Infinite Play” mode and can no longer level up.

Three exercises in each body region are unlocked at the beginning of use. These exercises were judged to be the easiest by an experienced occupational therapist who helped design the exercises. The software will unlock more difficult exercises if the cumulative level (of the currently unlocked exercises in the region) exceeds a threshold (5, then 10, 15, 20, 25, 30, 40, 50).

For example, if a new user interacts with the system for the first time, all of their exercises start at level 0. Then, if they start their first session in the arms region and complete the target number of repetitions 5 times in a row for one exercise and then five times in a row for a different exercise, their cumulative level in the arms region will be 10. After this session ends, the software will unlock two new exercises because the user passed both the 5 and 10 cumulative level thresholds.

During an exercise, the user has a set amount of time to complete the exercise, which is visually represented by a falling bar on the screen. Additional time is added for each repetition completed, but the rate at which the bar falls also increases over time, creating a gamified experience. Thus, if a user stops exercising or performs repetitions too slowly, time will run out (i.e., the bar will reach the bottom of the screen), and the exercise will end before the user was able to achieve the target number of repetitions.

As users typically acquire the system directly and use it on their own without supervision from a therapist, they are prompted to “take a tour” of the software when they first interact with the system. If the user chooses to do this, the system presents a sequence of messages explaining the graphical interface to perform their first exercise. The system also comes with a written user's guide and online resources (e.g., setup videos hosted on YouTube). For each exercise available in the system, there are written instructions and an embedded video users can access in which an occupational therapist explains the exercise and provides tips to properly complete the exercise. During the exercise, the interface presents a visual of the desired start and stop state for each repetition with a slider prompting the user to move between the two states (Figure 1, Bottom row, right).

### Data Acquisition and Cleaning

We used FitMi user data acquired between June 20th, 2016 and December 15th, 2019. We removed data from test users, clinic users, and users whose first exercise was <8 weeks prior to the end of our data collection (as described in the Supplemental Material), resulting in data for 2,581 users. We assigned the start time of each user's 1st day to be 12:00 am, counting days of use as 24-h periods after this start time.

We found that the total number of repetitions performed by each user during their first 8 weeks had a lognormal distribution (see Results). Thus, we filtered outliers from the data using the log transform of total repetitions performed, excluding users with log transformed data more than two standard deviations from the mean. This filter resulted in the exclusion of 117 (4.5 %) users with total repetition counts that were ≤ 16 or ≥79,380. A total of 2,464 users remained after this outlier removal process.

### Data Analysis

#### Estimating Upper Extremity Impairment

The large data set was anonymous and contained no clinical information about the users' impairment. We hypothesized that the rate of repetition of an exercise would reflect the user's motor impairment level. To test this hypothesis, we used data from a randomized controlled trial (RCT) using the FitMi system (ClinicalTrials.gov Identifier: NCT03503617) for which a rehabilitation therapist monitored 41 persons with a chronic stroke as they played three (out of the ten total) exercises in each of the four FitMi regions. The therapist also evaluated each participant with the Upper Extremity Fugl-Meyer (UEFM) assessment, a widely-used and validated measurement of upper extremity impairment after stroke that varies from 0 (meaning complete paralysis) to 66 (normal arm movement) (26). As described below, we studied the relationship between the subject's initial repetition rate for various FitMi exercises and their UEFM score.

#### Success Rate

A FitMi user “levels up” when they complete the target number of repetitions for a given exercise in the allotted time. We defined each user's “Success Rate” as the percentage of exercises in which the user leveled up during the 1st week of use, divided by the number of exercises they attempted during that 1st week, excluding Level 10 exercises because leveling up was not possible at Level 10.

#### Lifetime

**Figure 2:**
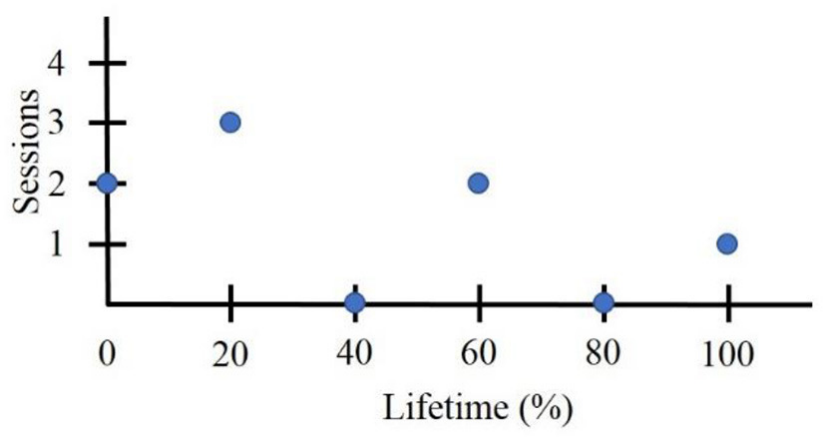
Sample lifetime data for a user who quit using FitMi after 6 days and initiated an exercise session only on 4days.

To compare trends between users over the entirety of their interaction with FitMi (i.e., beyond the eight-week window), we calculated various outcomes measures as a function of “lifetime.” We defined each user's lifetime as the period of time between their first (assigned value 0%) and final day of interaction (assigned value 100%). Data for all other days in the user's lifetime were proportionally distributed throughout the percent lifetime into 100 bins, each representing an increment of 1% (Figure 2). If a user had more than 100 days of activity, neighboring day data were summed and placed into the nearest 1% bin. When we calculated ensemble statistics across percent lifetime, we considered only bins corresponding to days on which the user could have used the system. We excluded users who used the system for only 1 day from the lifetime analysis, leaving 2,033 users for these analyses.

#### Steadiness of Use

**Figure 3:**
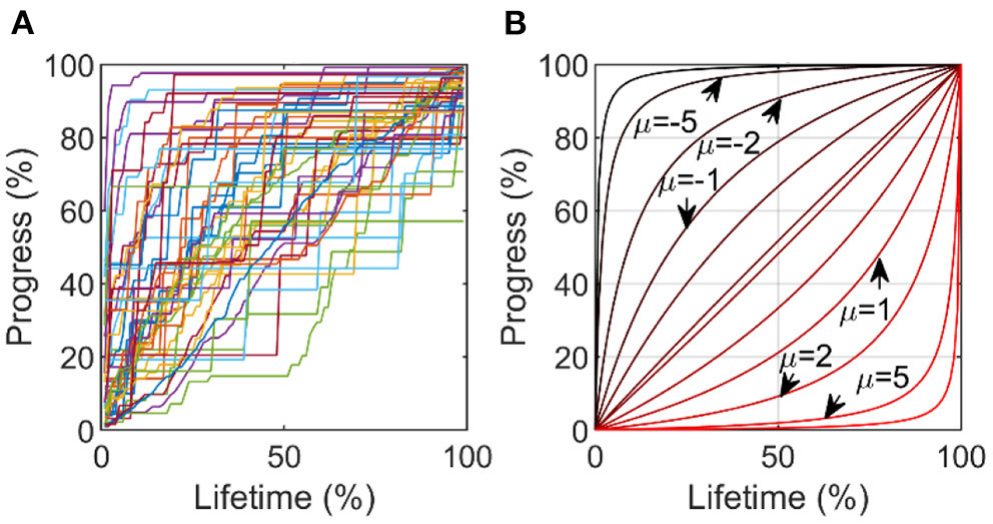
Estimating steadiness of use. **(A)** % Progress in exercises achieved vs. % lifetime. **(B)** We fit curves of this form, identifying the parameter μ as the “steadiness curvature.” A curve with μ = 0 indicates perfectly steady use over the lifetime, while a curve with μ = −5 indicates high initial use tapering rapidly off (deceleration), and a μ = 5 indicates light initial use, rapidly increasing (acceleration).

To quantify steadiness of use of the system, we first plotted the cumulative percentage of total exercises initiated vs. the percent lifetime (Figure 3A). We found that the curvature of the progress lines between users varied from concave to convex. We fit a function based on the smooth approximation from (27) with a single parameter μ (which we will refer to as the “steadiness curvature”) that specifies concavity, using the fmincon solver on MATLAB R2019b (Figure 3B). See Supplemental Material for function and details. We did not include the values of the first and last exercise session because these were always 0 and 100% and the fit curve also was constrained to have these values. We only included users with five or more active days in this analysis, to ensure there was sufficient data to estimate a curvature, resulting in 1,385 users.

#### Probability of Perseverance

We quantified perseverance as the probability that individuals would achieve low, medium, or high levels of usage. We defined the thresholds for low, medium, and high levels to be the 25th, 50th, and 75th percentiles of various measures of usage – total repetitions, total usage time, and total active days. We estimated the probability of perseverance for a given range of three factors (estimated impairment, success level, and steadiness of use) by finding all individuals within that range for that factor, then calculating the fraction of those individuals who exceeded the level of usage.

#### Statistical Tests for a Maximum in Perseverance

For the three factors we analyzed, measures of perseverance often had a maximum at an intermediate value of the factor and fell off in either direction from that value. To test whether this maximum was significant, we compared the maximum to the value immediately next to it (Phi test) or, if there were at least four neighboring values, we tested for a relationship in the descending region using regression analysis. For brevity, we will refer to this statistical methodology as the “sweet spot test” below, providing the 2 *p*-values needed to assess whether there was a significant declining trend on the left and right side of the peak, respectively.

## Results

### Usage Statistics

To provide context for the ensuing analysis of factors that predicted perseverance, we first provide summary statistics for usage of the system over the entire 3.5 year data snapshot window. The 2,464 users performed a mean of 245 ± 617 (SD) exercises per user, which they achieved over 16 ± 35 days of use. Users focused more on performing upper extremity exercise (39.9% of exercises were for the arms and 27.8% for the hands) vs. the core (15.8%) or leg (16.5%) exercises (see Supplementary Figure S1). The average time spent on each exercise was 58.2 ± 52.7 s, resulting in a mean total exercise time of 237 ± 682 min (3.9 h), during which users achieved 13,033 ± 42,789 repetitions.

**Figure 4:**
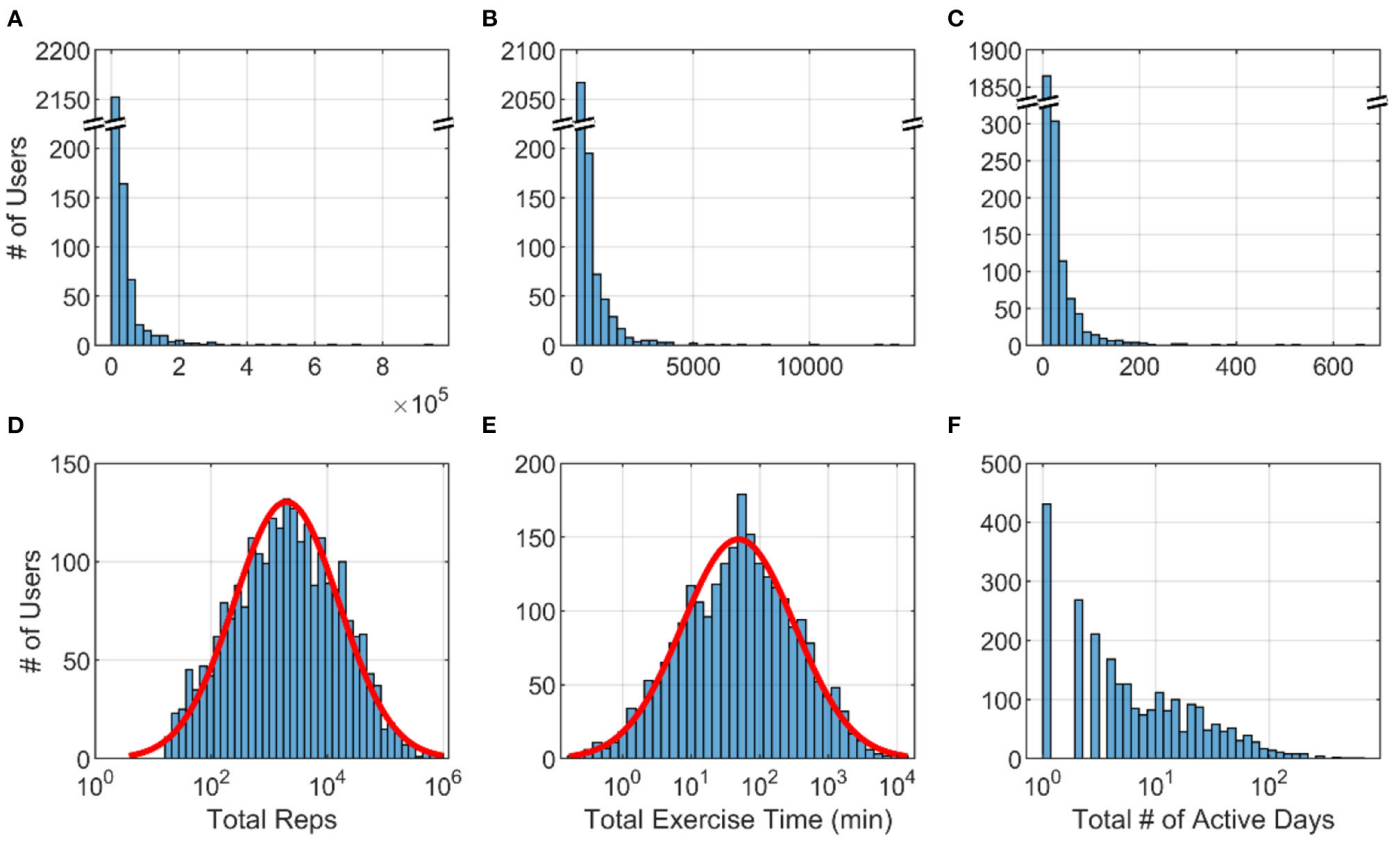
Histograms across 2,464 FitMi users for **(A)** total repetitions **(B)** total exercise time and **(C)** total active days. **(D–F)** show the same data plotted on a log scale for the x-axis. Red lines are best-fit normal distributions.

Total repetitions and total exercise minutes (but not total # of active days) were distributed in log-normal fashion (Figure 4). Thus, while there were a large number of users who used the system only lightly and relatively fewer heavy users, there was no clear demarcation between them. The top 1% of users completed a mean of 332,189 repetitions, a ~25-fold increase in perseverance compared to the average user.

### Factors Associated With Perseverance

#### Impairment Level

**Figure 5:**
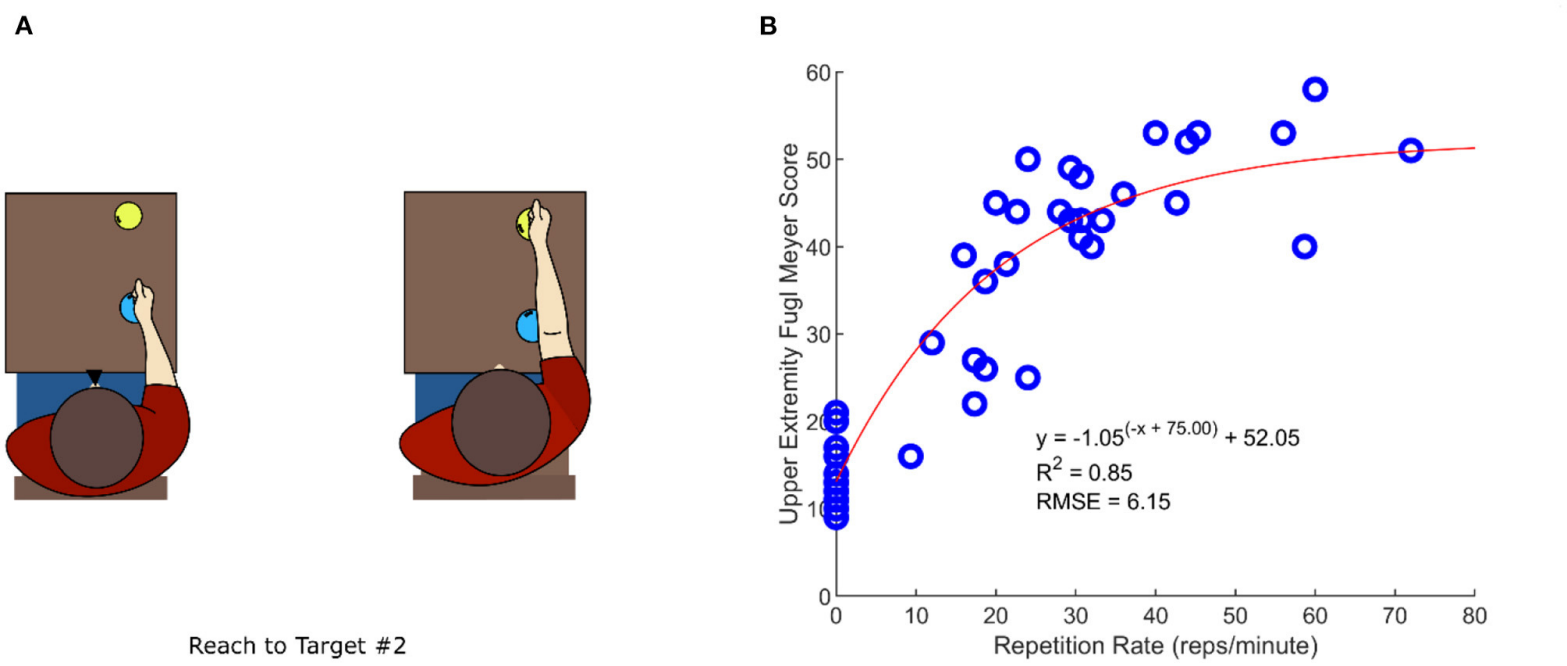
Identifying a system measurement that relates to clinically-measured, upper extremity impairment. **(A)** “Reach to Target #2” exercise diagram **(B)** Relationship between the Upper Extremity Fugl-Meyer (UEFM) score, assessed by a rehabilitation therapist, and repetition rate of the “Reach to Target #2” exercise. Shown is an exponential curve fit with the associated statistics.

To determine a sensor-based measurement that we could associate with impairment, we first analyzed the data acquired from 41 individuals with hemiparesis after stroke who used FitMi in a clinic under the supervision of a rehabilitation therapist. Among the six upper-extremity FitMi exercises tested, the initial repetition rate of the “Reach to Target #2” exercise was most strongly correlated with UEFM score (adjusted *R*^2^ = 0.75, *p* < 0.001), a common clinical measure of upper extremity impairment. The relationship was well-fit by an exponential function (Figure 5).

**Figure 6:**
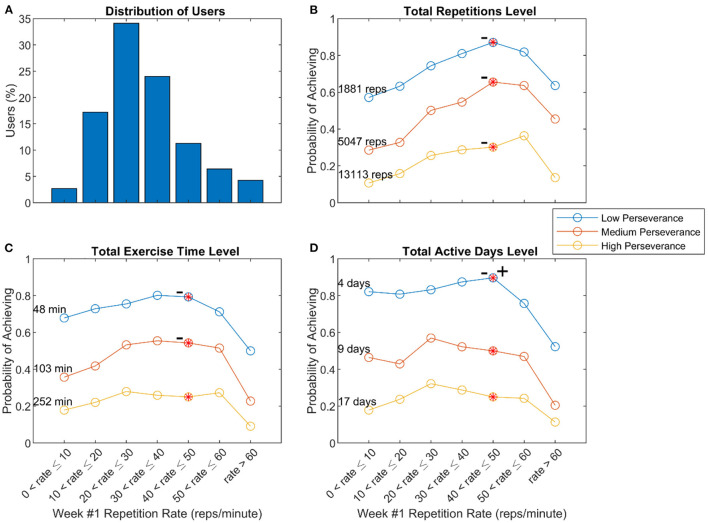
Relationship of repetition rate of the Reach to Target # 2 exercise in Week 1 and perseverance. **(A)** Distribution of users with various repetition rates **(B–D)** Probability of achieving low, medium, and high levels of perseverance, defined to be the 25th, 50th, and 75th percentiles of three measures of usage – total repetitions **(B)**, total exercise time **(C)**, and total active days **(D)** – measured across 8 weeks of use. The – symbol indicates a significant decline from the peak value moving to the left, and + sign indicates a significant decline from the peak value moving to the right, using the “sweet spot” test described in the methods (*p* < 0.05).

This repetition rate measured in the 1st week predicted perseverance, measured as the probability of achieving various levels of either total repetitions, exercise time, or active days during Weeks #1–8 (Figure 6). Users with lower repetition rate (i.e., greater estimated impairment) exhibited a perseverance probability that was decreased by 7–64% compared to users with the maximum probabilities, although this trend was not always significant. Users with higher repetition rate (i.e., lower impairment) also exhibited probabilities decreased by 27–64%, but this trend was significant only for the active days measure. The optimal range of 40–50 reps/min indicated that people with generally less impairment tended to persevere more, however, and corresponded to a relatively mild UEFM score (Figure 5B).

#### Success Level

**Figure 7:**
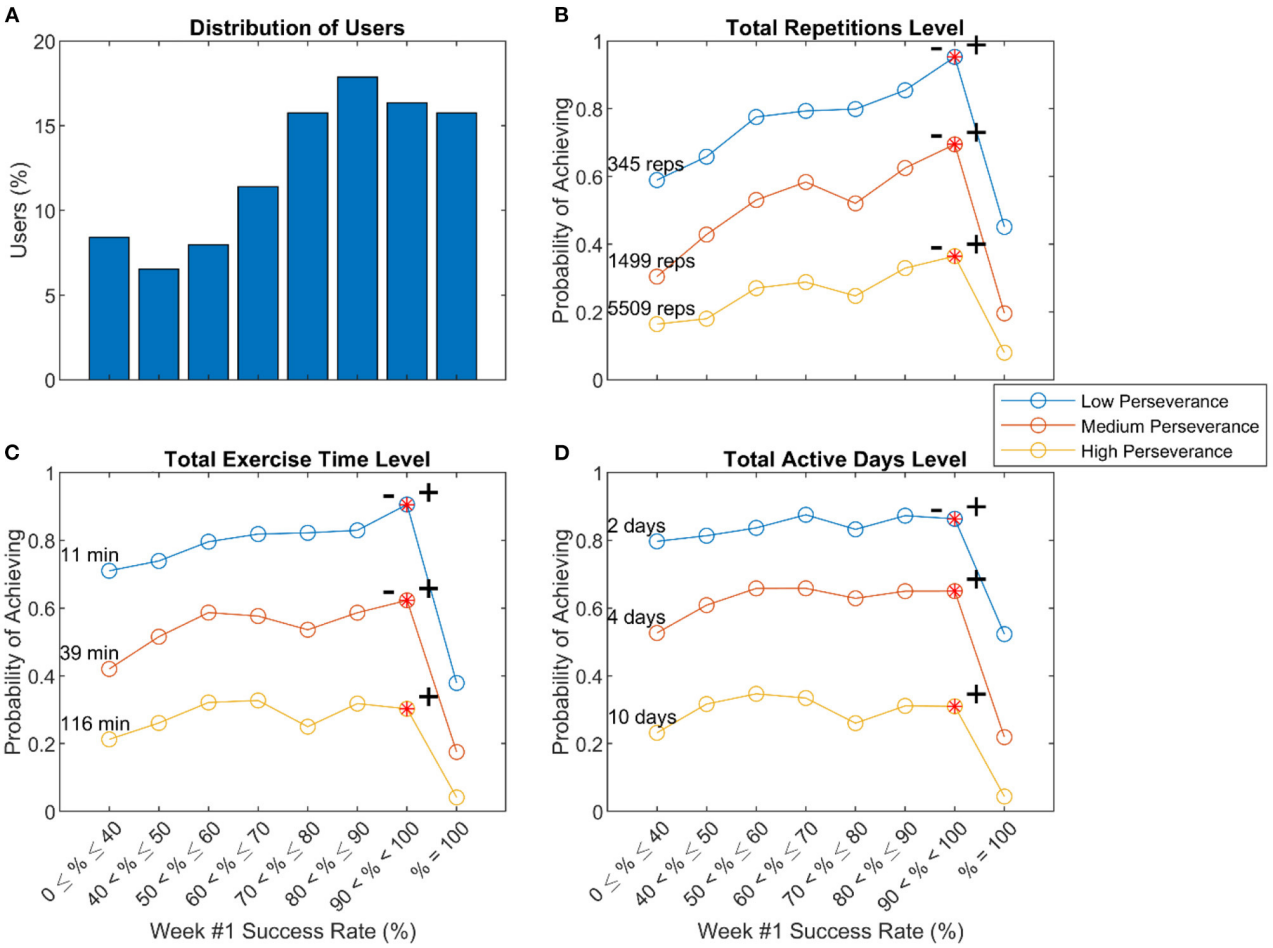
Relationship of initial success and perseverance. **(A)** Distribution of users with various success levels in leveling up during Week #1 **(B–D)** Probability of achieving low, medium, and high levels of perseverance, defined to be the 25th, 50th, and 75th percentiles of three measures of usage – total repetitions **(B)**, total exercise time **(C)**, and total active days **(D)** – measured across 8 weeks of use. The – and + symbols indicated significant declines to left and right, respectively, from the peak value using the “sweet spot” test described in the methods (*p* < 0.05).

People who experienced lower levels of success in the 1st week of use exhibited decreased probabilities of achieving the different levels of use in the 8-week window, as did people who experienced 100% success (Figure 7). Thus, there was an optimal range of initial success associated with perseverance, which was above 90 but not 100%. Notably, only 45% of users who achieved 100% success rates initiated another exercise after the 1st week, while 72% of users who achieved lower success rates initiated an exercise after the 1st week, a significant difference (Phi Test, Coeff = 0.2, *p*-value < 0.001). About 85% of users who obtained 0% success rates went on to initiate another exercise after the 1st week.

#### Steadiness of Use

**Figure 8:**
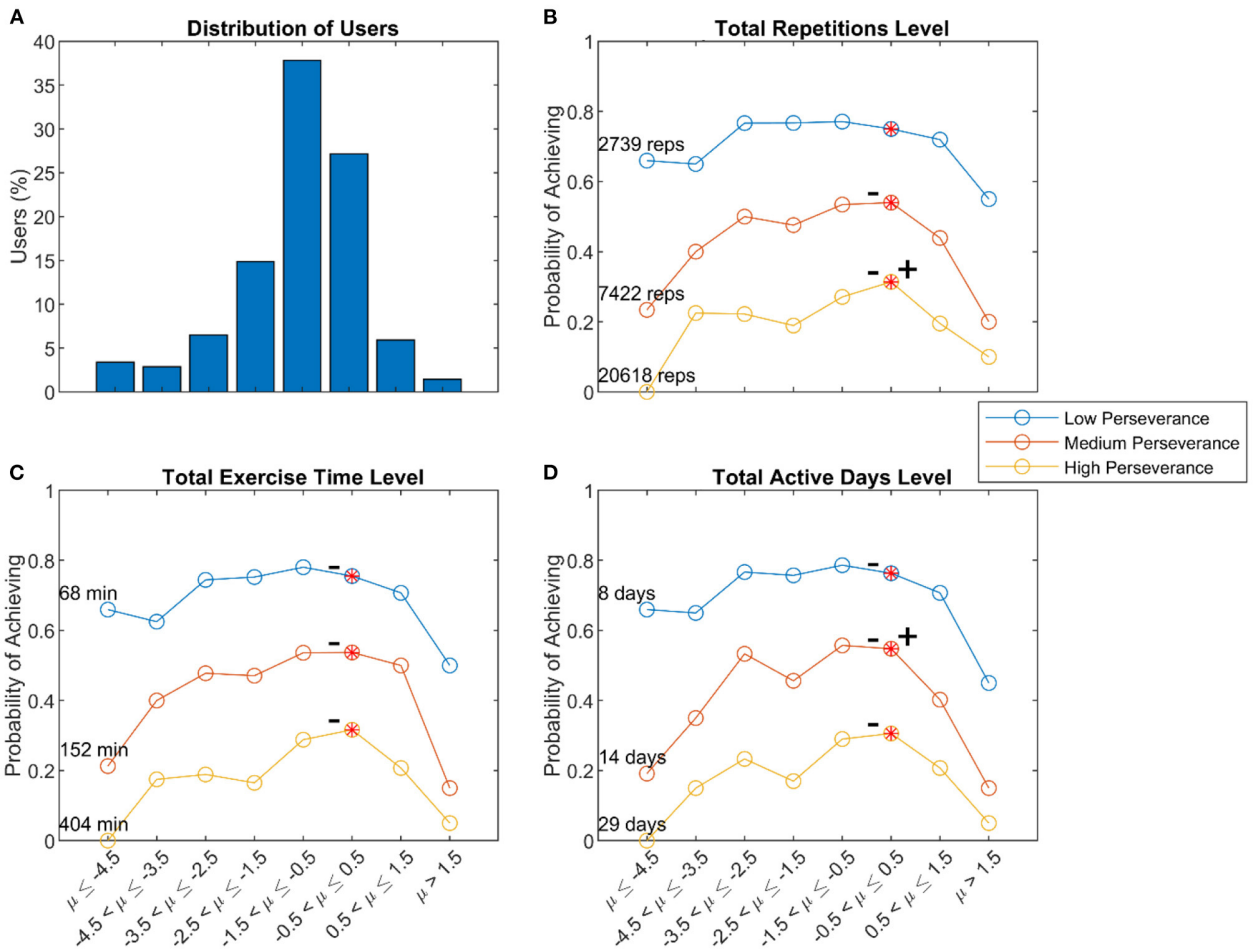
Relationship of steadiness curvature and perseverance. **(A)** Distribution of users with various steadiness curvatures. Probability of achieving low, medium, and high levels of perseverance, defined to be the 25th, 50th, and 75th percentiles of three measures of usage – total repetitions **(B)**, total exercise time **(C)**, and total active days **(D)** – measured across 8 weeks of use. The – and + symbols indicated significant declines from the peak value to the left and right, respectively, using the “sweet spot” test described in the methods (*p* < 0.05). In the above analysis, user's initial repetition rates, success rates, and steadiness curvature included data from Week #1, and we used them to predict overall perseverance, which also included data from Week #1. To test if including data from Week #1 biased the patterns we observed, we repeated the above analyses with measures of perseverance calculated over Weeks #2–8. The results of these analyses were similar to the results presented above.

Some users initiated exercises at a steady rate over time, while others exercised at a high-then-low rate (decelerating), or a low-then-high rate (accelerating). The steadiness curvature (μ from Figure 3) had a mean curvature value of −1.1, which was significantly <0 (*t*-test, *p* < 0.01) (Figure 8A). Users with greater steadiness curvature (either decelerating or accelerating) tended to use the system less (Figure 8). Thus, there was an optimal range for steadiness of exercise initiation associated with the probability of perseverance, although the relationship was relatively flat in the intermediate range.

### Session Initiation Probability

**Figure 9:**
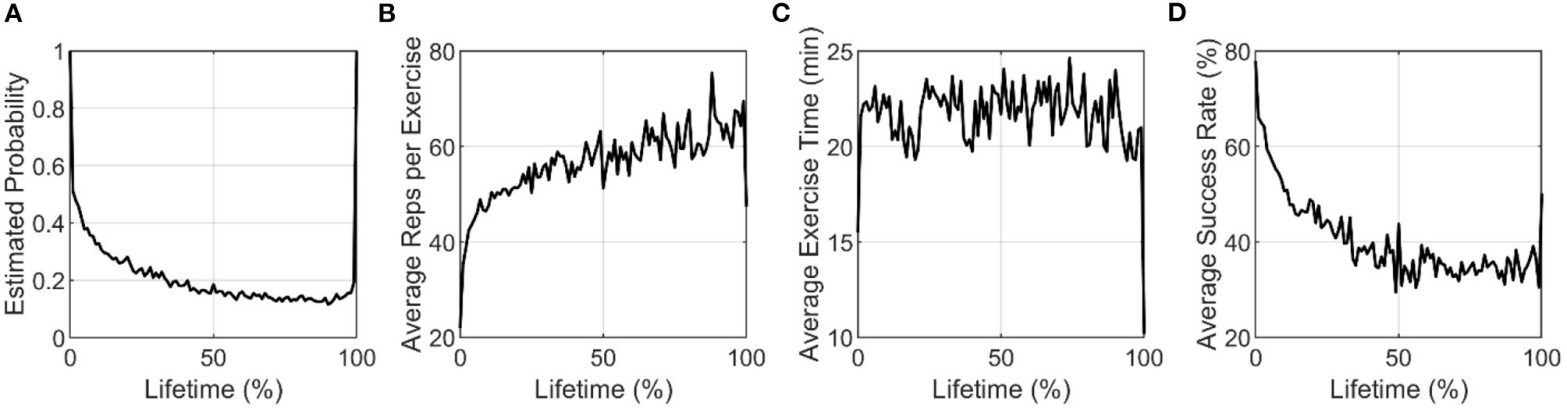
**(A)** Estimated probability of initiating at least one session, and **(B–D)** Average activity metrics plotted against percent lifetime for active users only. By our definition of percent lifetimes, all users are active on the first and last ticks of the normalized lifetimes.

The results of the analysis of steadiness of use indicated that users on average had a decelerating pattern of use (since the mean steadiness curvature was −1.1). We studied this decelerating pattern further by calculating the probability of initiating a session across the population as a function of lifetime. On average across the population, session initiation probability decreased in an exponential-like fashion over the lifetime of use (Figure 9A). However, when users initiated an exercise, on average they achieved increasingly more exercise repetitions over time (Figure 9B). The amount of time spent exercising *per session* stayed roughly constant (around 20 min, Figure 9C), so the increase in repetitions was attributable to doing more repetitions per minute, a demand the game software automatically imposed on users as they “leveled up.” As a result of this demand, users had less success leveling up over time (Figure 9D).

**Figure 10:**
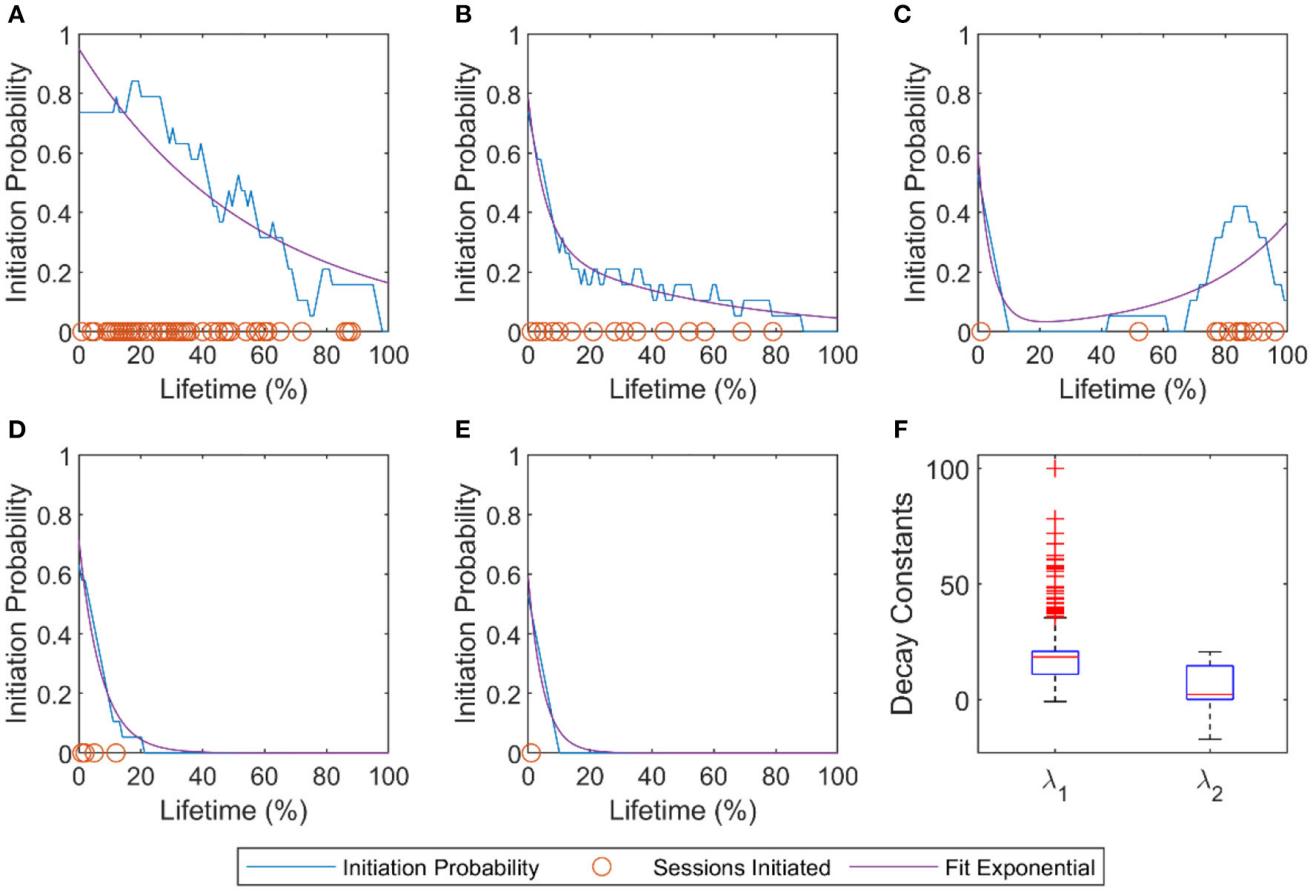
Pattern of individual users' session initiation probabilities. **(A–E)** Examples of five users' session initiation probability demonstrating a variety of the usage patterns. Orange circles mark initiation of at least one exercise session at that lifetime %. The blue line is the probability of initiating at least one session calculated with a sliding window average (window size = 19). The purple line is the sum of two exponential functions fit to each user's probability of session initiation curve. **(F)** Decay constants of the two exponentials. For the model fit, $f\left(x\right)=ae^{-\lambda_{1}x}+be^{-\lambda_{2}x}$, the average coefficients of the population were *a* = 0.42±0.17, λ_1_ = 16.7±9.3, *b* = 0.43±0.17, λ_2_ = 6±8.7.

We observed an exponential-like decrease in session initiation probability on average across the user population. To determine if individuals also tended to follow exponential-like decreases in session initiation probability, we tested the ability of various decay functions (the sum of two exponential functions, a single power function, and a double power function) to fit individual users' session initiation probability curves plotted over their lifetime. For individual users, we estimated session initiation probability (for one or more sessions in a day) using a moving average with a window of size 19% of lifetime, padding the start and end of the data with ones and zeros, respectively. The value of 19% was chosen to give a smooth curve; smaller or larger windows did not alter the main results. Only users with more than 1 day of activity were fit. The sum of two exponential functions fit users significantly better than the other two with an average adjusted *R*2 of 0.84 (0.19 SD) vs. 0.50 (0.13 SD) and 0.70 (0.19 SD) for the single and double power functions, respectively. Fit statistics for different functions were significantly different (Kruskal Wallis *p* < 0.001). Thus, session initiation was best characterized by the sum of two exponential decay processes (Figure 10). The decay constants, λ, were on average 16.7 (9.3 SD) and 6 (8.7 SD). Converting the decay constants to time constants, τ (where $\tau =\frac{1}{\lambda }$), shows these processes had an average mean time constant of 8.8% (2.9 SD) and 51.6% (17.3 SD) of normalized lifetime, respectively.

## Discussion

The data set studied here represents the self-determined exercise patterns of a large number of individuals continuing their rehabilitation at home without formal supervision following purchase of a commercial exercise technology. We tested whether three factors—impairment level, initial success, and steadiness of use—were associated with persevering at home rehabilitation exercise over an eight-week window. For each factor there was an optimal range associated with higher perseverance: mild impairment (but not too mild), high levels of success (but not perfect success), and steady, regular use, respectively. We also observed that the amount of exercise being achieved decelerated over time. This deceleration was attributable to an exponentially decreasing probability of initiating sessions, rather than a declining amount of exercise within a session. We first discuss the relevance of these findings for home rehabilitation exercise programs and then limitations and directions for future research.

### Perseverance and Motor Impairment

Severity of motor impairment would be expected to predict perseverance because people who are weaker and less coordinated perceive exercise as more effortful and difficult (28). Since we did not have any clinical information about the users, we identified a sensor-based measure of impairment level – repetition rate of the Reach to Target #2 exercise. This rate strongly correlated with a widely used clinical measure of upper extremity impairment (the UEFM score), as measured in a separate, clinically-monitored study (ClinicalTrials.gov Identifier: NCT03503617).

There was a downward trend in probability of achieving various levels of total repetitions, minutes of use, and active days of exercise as repetition rate decreased; this trend was significant in six of the nine curves considered (Figure 6). This trend is expected when the total number of repetitions is the perseverance measure (since exercising at a slower repetition rate should produce lower total number of reps), but it was also at least partially true when total minutes of use and total days of exercise were the measures, which are behavioral decisions rather than performance related. This finding supports the concept that people who are more severely impaired have marginally greater difficulty persevering with home rehabilitation exercise.

However, this was not a strong effect, as not all curves examined had a statistically significant maximum despite the large sample size. This may be due in part to the fact that the FitMi system was designed to accommodate people with a wide range of impairment levels by including exercises with a broad range of difficulties, including exercises with simple movements and low requirements for hand dexterity.

### Perseverance and Optimal Challenge

A striking observation about both the impairment and initial success factors was that perseverance increased steadily as capability increased, but then fell off dramatically near the highest level of capability. This finding is consistent with the idea that training should be “not so hard that we are discouraged, but not so easy that we get bored” (29).

The Challenge Point Hypothesis is influential in motor learning theory and posits that the amount of learning will be maximized when the challenge presented during practice is optimized (16, 30). Several studies suggest, however, that the self-selected amount of practice is also maximized when the challenge is optimized. For example, rats who were given a running wheel to run on in their cage at night ran less when the wheel incorporated more resistance (31). Reducing success at playing a video game during robotic hand rehabilitation exercise after stroke lowered motivation and self-efficacy (19). Persons with a stroke engaging in technology-aided home rehabilitation seem to intuit this principle. In a recent study of a wearable sensing glove for practicing finger dexterity, home users tended to select difficulty parameters so that they practiced the game at a high success rate (near 90%) (32). To achieve this, they adjusted difficulty up and down based on their recent experience of success or failure. Success rates of ~80–90% have been found to optimize learning for a broad class of learning algorithms that are useful in describing human and animal perceptual, motor, and reinforcement learning (29). The results of this study contribute to the principle that 80–90% success is desirable for promoting learning-related activity in terms of practice dosage.

This notion is also consistent with Csikszentmihalyi's concept of flow—an enjoyable psychological state that occurs when individuals are engaged in optimal challenges (33). Flow theory posits that an activity becomes intrinsically rewarding (hence, more likely to be initiated and sustained) when the individual's skillset is appropriate for the task but continues to develop, and the level of challenge is gradually increased. Our findings support what others have proposed—incorporating elements that optimize flow into design games for rehabilitative therapy could be helpful for promoting adherence and optimizing outcomes (34, 35).

### Perseverance and Steadiness of Use

Steadiness of use was also associated with perseverance. Users with heavily accelerating steadiness curvatures often had substantial periods of inactivity shortly after they first started using FitMi, followed then by periods of increased use. On the other hand, heavily decelerating users tended to exhibit less usage as well. They may have experienced burnout from exercising too much too quickly, becoming fatigued or sore from exerting themselves, or become bored with the system, consistent with a novelty effect. Factors unique to FitMi might have contributed to decreasing session initiation probability as well. For example, after leveling up, users could not practice at previously experienced levels of an exercise. Thus, users eventually reached levels where they could no longer achieve high success rates. While this design choice forced users to perform exercises at higher repetition rates over time, the increasing game failure rates may have decreased session initiation probability.

We highlight the finding that it was the decreased session probability, rather than the decreased amount of exercise within a session, that was associated with reduced perseverance. That is, it was the act of “getting started again” rather than “finishing a session” that users struggled with. Indeed, the smaller and larger time constants found in the analysis of individuals' initiation decrease may reflect two processes, a brief period of higher use due to a novelty effect (25) and a lower use period with slower decay due to the individual's intrinsic motivation, an important clue for rehabilitation technology developers we discuss further below.

### Information Contained in the Distribution of Usage

Total repetitions and exercise time were distributed log-normally. Thus, a large portion of users had low perseverance, while a small portion of users performed substantially more than their peers (recall that the top 1% of users used the system ~25 times more than the average user). It is important to notice, however, that the data were distributed smoothly, preventing clear clustering of users into low, moderate, or high performing users. Log-normal distributions arise in many branches of science, including analyses of human behavior (36) and characterizations of neurophysiological parameters (37), because they occur in samples where the mean value is low, the variance is large, and the data cannot take negative values (36), conditions met in the present study. Mechanistically, log-normal distributions arise as the result of a multiplicative effect of small independent factors, such as repeated choices (36, 38). For the population we studied, it may be that the multiplicative effect of the daily choices the users made on whether or not to initiate a session caused a log-normal distribution of perseverance to arise (32). If so, another important clue for rehabilitation technology developers provided by this analysis is to consider various means to influence daily choices that users make to exercise or not.

### Limitations and Future Work

There are several potential limitations of this work. The data in this study are anonymous, and we did not have access to any demographic or clinical data about users beyond their direct interactions with the system. For example, our measure of impairment was inferred from exercise rates and only partially characterized impairment. Biological, psychological, and socio-environmental measures are key predictors of health outcomes (39), as well as rehabilitation outcomes for people who have had a stroke (40–42). Due to the anonymity of the data, our analysis did not include any biopsychosocial measures, and this limits our ability to compare results with other work on stroke. It is also important to note that our results are likely biased toward a specific type of person, limiting their generalizability. Namely, we studied people who: (a) could afford the technology and had internet access (which allowed the anonymous data collection); (b) had a profile of disabilities that did not inhibit them from engaging with the system (e.g. sufficient visual and cognitive capabilities); and (c) were motivated enough to seek out a technology to continue rehabilitation. As FitMi is marketed for stroke rehabilitation, we assume that most users have experienced a stroke, but other types of users undergoing rehabilitation were possible (e.g., people with spinal cord injury or cerebral palsy), and this may have introduced further variability. An important direction for future research is to understand the psychological, demographic, and clinical characteristics of FitMi users that also modulate perseverance (43), potentially through surveys sent to FitMi users. A small fraction of data entries for a given user may have been generated by other people with access to the device playing with the system, or by a clinical facility purchasing the home version of FitMi and using a single user ID with multiple patients. We filtered potentially anomalous users to limit their impact. Despite these limitations, we believe the present study provides a first-of-its-kind demonstration that inferences about perseverance with rehabilitation exercise can be drawn even from anonymous patterns of sensor usage. Of note, analyses drawn from anonymous usage patterns maintain privacy while also providing insights into how to optimize home rehabilitation systems.

A key principle supported by our analysis is that providers of home rehabilitation exercises should design programs that appropriately challenge the participant. High levels of success are desirable, but 100% success rates should be avoided. The company that produces FitMi has already implemented software changes based on these findings. The changes allow less successful users to increase their success, and highly successful users to increase their challenge more quickly. Another approach could be to incorporate an initial assessment of user capability into the home exercise system, and then to titrate challenge based on the assessment results.

Our findings also suggest that providers of home rehabilitation exercises should incorporate methods to sustain session initiation. Beyond factors intrinsic to the exercises such as challenge, extrinsic factors, such as providing reminders to exercise (44), having a therapist monitor achieved amounts of exercise (45), encouraging the involvement of caregivers (46), promoting self-efficacy through actions plans or goal setting (47), or embedding the exercise program in a supportive community (48) will also likely play a critical role in making home rehabilitation effective and could be examined in future studies with FitMi.

Other important directions for future research include the following. It is feasible to conduct large-scale experiments to test factors that influence perseverance—such as challenge level or other factors—by releasing modified versions of the software to subgroups of users; indeed, as mentioned above, the company that produces FitMi has already implemented a new version of the FitMi software based on the findings of this study, and as users accumulate, it will be possible to test whether these changes improved perseverance. It is also feasible to quantify the exact amount of exercise associated with generating a therapeutic benefit on movement capability, which would help settle an open, fundamental question in rehabilitation practice (49, 50). Using artificial intelligence to predict dropout and to automatically send encouragements or adapt system parameters is an interesting possibility (51). Finally, an important direction for future research is to study the “super-users” who persevered the most to try to determine what factors contributed to their extreme behavior. Perhaps by understanding the motivational characteristics of super-users, strategies could be developed to help other users better persevere in their rehabilitation.

## Data Availability Statement

The data analyzed in this study was obtained from Flint Rehabilitation Devices, LLC, the following licenses/restrictions apply: Requests to access these datasets must be granted by Flint Rehabilitation Devices, LLC. Requests to access the datasets should be directed to DZ, dzondervan@flintrehab.com.

## Ethics Statement

The studies involving human participants were reviewed and approved by UC Irvine Institutional Review Board. Written informed consent from the participants' legal guardian/next of kin was not required to participate in this study in accordance with the national legislation and the institutional requirements.

## Author Contributions

DR, DZ, GC, and ER contributed to the conception and design of the study. DZ provided the data. ER curated the data for analysis and wrote the first draft of the manuscript. ER, VS, GC, and CJ performed analysis. DZ, RA, AR, and DR provided input and feedback on the data analysis and interpretation. DR, VS, and CJ wrote sections of the manuscript. All authors contributed to manuscript revision, read, and approved the submitted version.

## Funding

This research was supported by the ICT Access for Mobile Rehabilitation (mRehab) Rehabilitation Engineering Research Center, National Institute of Independent Living, Disability, and Rehabilitation Research, 90REGE0011.

## Conflict of Interest

DR has a financial interest in Hocoma A.G. and Flint Rehabilitation Devices LLC, companies that develop and sell rehabilitation devices. Flint Rehabilitation Devices produces the FitMi sensor used in this study. The terms of these arrangements have been reviewed and approved by the University of California, Irvine, in accordance with its conflict-of-interest policies. DZ has a financial interest in Flint Rehabilitation Devices, LLC. The remaining authors declare that the research was conducted in the absence of any commercial or financial relationships that could be construed as a potential conflict of interest.

## Publisher's Note

All claims expressed in this article are solely those of the authors and do not necessarily represent those of their affiliated organizations, or those of the publisher, the editors and the reviewers. Any product that may be evaluated in this article, or claim that may be made by its manufacturer, is not guaranteed or endorsed by the publisher.

## Supplementary Material

### Data Cleaning

Users with the following characteristics were removed from the data set:

- User accounts created before July 1st, 2017 (when FitMi became commercially available), because these users were test users.
- Users with “clinic” or “generic” installations of FitMi, because this work is focused on home rehabilitation. Users with “home” installations were kept.
- Users labelled as a “Super User” and/or “Staff”.
- Users with entries from other Flint Rehab software, because they are likely test users.
- Users with multiple/inconsistent Install Type values, because they are likely test users.
- Users that reverted from the new/current version of the FitMi software to the old version (this only occurred with one user).
- Users that accessed the FitMi software but never performed any exercises.
- Users with region and exercise name entries not found in commercial software, because they are likely test users.
- Users that owned the FitMi software for less than eight weeks since their first exercise, to exclude users that had recently bought FitMi.
- Users whose first exercise did not start at level zero, because they have data that is either missing or corrupted.

### Steadiness Curvature Function

Our steadiness curvature function was based on the smooth approximation function, $f\left(x\right)=\sqrt{x^{2}+C}$, from (27). Various modifications and transformations were used to ensure that our steadiness curvature function started at 0 and ended at 100, while maintaining symmetry across the diagonal (i.e., *y* = −*x*+100 in Figure 3).

Supplementary Figure S1 Percent distribution of all exercises done in the **(A)** Arms, **(B)** Core, **(C)** Legs, and **(D)** Hands regions by users in the study. The exercises for each region are plotted in order of difficulty from left to right. Initially the first three exercises of each region are unlocked. As the user levels up, exercises are unlocked in order of increasing difficulty.

## References

[1] Cieza A Causey K Kamenov K Hanson SW Chatterji S Vos T. Global estimates of the need for rehabilitation based on the global burden of disease study 2019: a systematic analysis for the global burden of disease study 2019. The Lancet. (2020) 396:2006–17. 10.1016/S0140-6736(20)32340-0 33275908 PMC7811204

[2] Godwin KM Wasserman J Ostwald SK. Cost associated with stroke: outpatient rehabilitative services and medication. Topics Stroke Rehabil. (2011) 18:676–84. 10.1310/tsr18s01-676 22120036

[3] Ottenbacher KJ. Trends in length of stay, living setting, functional outcome, and mortality following medical rehabilitation. JAMA. (2004) 292:1687. 10.1001/jama.292.14.1687 15479933

[4] Kilova K Kitova T Kasnakova P. Telemedicine in help of rehabilitation in the conditions of COVID-19. Health Policy Technol. (2021) 10:100508. 10.1016/j.hlpt.2021.100508 33850698 PMC8026262

[5] Dantas LO Barreto RP Ferreira CH. Digital physical therapy in the COVID-19 pandemic. Br J Phy Therapy. (2020) 24:381–3. 10.1016/j.bjpt.2020.04.006 32387004 PMC7252186

[6] Malec JF Salisbury DB Anders D Dennis L Groff AR Johnson M. Smith, response to the COVID-19 pandemic among post-hospital brain injury rehabilitation providers. Arch Phy Med Rehabil. (2021) 102:549–55. 10.1016/j.apmr.2020.10.137 33253694 PMC7695439

[7] De Biase SL Cook DA Skelton M ten Hove WR. The COVID-19 rehabilitation pandemic 1. Age Ageing. (2020) 49:696–700. 10.1093/ageing/afaa118 32470131 PMC7314277

[8] Bassett S. The assessment of patient adherence to physiotherapy rehabilitation. NZ J Physiotherapy. (2003) 31:60–6.

[9] Mourad S Eddine HK Karaki H Hassan KH. Patient's adherence to prescribed home exercises: Barriers and interventions. Gen Mol Res. (2018) 12:17. 10.4238/gmr16039898

[10] Peek K Carey M Mackenzie L Sanson-Fisher R. Patient adherence to an exercise program for chronic low back pain measured by patient-report, physiotherapist-perception and observational data. Physiotherapy Theory Pract. (2019) 35:1304–13. 10.1080/09593985.2018.1474402 29771180

[11] Holden MA Haywood KL Potia TA Gee M McLean S. Recommendations for exercise adherence measures in musculoskeletal settings: a systematic review and consensus meeting (protocol). Syst Rev. (2014) 3:10. 10.1186/2046-4053-3-10 24512976 PMC3923391

[12] Pugliese M Ramsay T Johnson D Dowlatshahi D. Mobile tablet-based therapies following stroke: a systematic scoping review of administrative methods and patient experiences. PLoS ONE. (2018) 13:e0191566. 10.1371/journal.pone.0191566 29360872 PMC5779660

[13] Zhou X Du M Zhou L. Use of mobile applications in post-stroke rehabilitation: a systematic review. Top Stroke Rehabil. (2018) 25:489–99. 10.1080/10749357.2018.1482446 30209991

[14] Neibling BA Jackson SM Hayward KS Barker RN. Perseverance with technology-facilitated home-based upper limb practice after stroke: a systematic mixed studies review. J Neuro Engin Rehabil. (2021) 18:43. 10.1186/s12984-021-00819-1 33627126 PMC7905577

[15] Adie K Schofield C Berrow M Wingham J Humfryes J Pritchard C. Does the use of Nintendo Wii SportsTM improve arm function? Trial of WiiTM in stroke: a randomized controlled trial and economics analysis. Clin Rehabil. (2017) 31:173–85. 10.1177/0269215516637893 26975313

[16] Brown DA Lee TD Reinkensmeyer DJ Duarte JE. Designing robots that challenge to optimize motor learning. in: Reinkensmeyer DJ Dietz V (Eds.), Neurorehabilitation Technology, Cham: Springer International Publishing (2016): p. 39–58. 10.1007/978-3-319-28603-7_3

[17] Zimmerli L Krewer C Gassert R Müller F Riener RL Lünenburger. Validation of a mechanism to balance exercise difficulty in robot-assisted upper-extremity rehabilitation after stroke. J Neuro Enginee Rehabil. (2012) 9:6. 10.1186/1743-0003-9-6 22304989 PMC3286404

[18] J.O. Wobbrock, J.O. Wobbrock, Improving Pointing in Graphical User Interfaces for People with Motor Impairments Through Ability-Based Design, Available online at: https://Services.Igi-Global.Com/Resolvedoi/Resolve.Aspx?Doi=10.4018/978-1-4666-4438-0.Ch008. (1AD). Available online at: https://www.igi-global.com/gateway/chapter/78429 (accessed August 6, 2021).

[19] Rowe JB Chan V Ingemanson ML Crame SC Wolbrecht ET Reinkensmeyer DJ. Robotic assistance for training finger movement using a hebbian model: a randomized controlled trial. Neurorehabil Neural Repair. (2017) 31:769–80. 10.1177/1545968317721975 28803535 PMC5894506

[20] McPherson KM Brander P Taylor WJ McNaughton HK. Consequences of stroke, arthritis and chronic pain—are there important similarities?. Disabil Rehabil. (2004) 26:988–99. 10.1080/09638280410001702414 15371047

[21] Ryan RM Deci EL. Self-determination theory and the facilitation of intrinsic motivation, social development, and well-being. Am Psychol. (2000) 55:68. 10.1037/0003-066X.55.1.68 11392867

[22] McAuley E Szabo A Gothe N Olson EA. Self-efficacy: implications for physical activity, function, and functional limitations in older adults. Am J Lifestyle Med. (2011) 5:92704. 10.1177/1559827610392704 24353482 PMC3864698

[23] Fu V Weatherall M McPherson K Taylor W McRae A Thomson T. Taking charge after stroke: a randomized controlled trial of a person-centered, self-directed rehabilitation intervention. Int J Stroke. (2020) 15:954–64. 10.1177/1747493020915144 32293236 PMC7739137

[24] Roger EM. Diffusion of Innovations. New York: Free Press (1995).

[25] Shin G Feng Y Jarrahi MH Gafinowitz N. Beyond novelty effect: a mixed-methods exploration into the motivation for long-term activity tracker use. JAMIA Open. (2019) 2:62–72. 10.1093/jamiaopen/ooy048 31984346 PMC6952057

[26] See J Dodakian L Chou C Chan VA McKenzie DJ Reinkensmeyer SC Cramer. A standardized approach to the Fugl-Meyer assessment and its implications for clinical trials. Neurorehabil Neural Repair. (2013) 27:732–41. 10.1177/1545968313491000 23774125

[27] Ramirez C Sanchez R Kreinovich V Argaez M. √(x2 μ) is the most computationally efficient smooth approximation to |x|: a proof, departmental technical reports (CS). (2013). Available online at: https://scholarworks.utep.edu/cs_techrep/789

[28] Reinkensmeyer DJ Housman SJ. “If I can't do it once, why do it a hundred times?”: Connecting volition to movement success in a virtual environment motivates people to exercise the arm after stroke. Virtual Rehabil. (2007) 8: 44–48. 10.1109/ICVR.2007.4362128

[29] Wilson RC Shenhav AM Straccia J Cohen D. The eighty five percent rule for optimal learning. Nat Comm. (2019) 10:4646. 10.1038/s41467-019-12552-4 31690723 PMC6831579

[30] Guadagnoli MA Lee TD. Challenge point: a framework for conceptualizing the effects of various practice conditions in motor learning. J Motor Behav. (2004) 36:212–24. 10.3200/JMBR.36.2.212-224 15130871

[31] Ishihara A Roy RR Ohira YY Ibata V Edgerton R. Hypertrophy of rat plantaris muscle fibers after voluntary running with increasing loads. J App Physiol. (1998) 84:2183–9. 10.1152/jappl.1998.84.6.2183 9609816

[32] Sanders Q Chan V Augsburger R Cramer SC Reinkensmeyer DJ Do AH. Feasibility of wearable sensing for in-home finger rehabilitation early after stroke. IEEE Transact Neural Sys Rehabil Engin. (2020) 1:1. 10.1109/TNSRE.2020.2988177 32305930 PMC9345607

[33] Csikszentmihalyi M. Flow: *The Psychology of Optimal Experience, 1st edition*. New York: Harper Perennial Modern Classics (2008).

[34] Constantinescu G Rieger J Mummery K. Hodgetts W. Flow and grit by design: exploring gamification in facilitating adherence to swallowing therapy. Am J Speech-Lang Pathol. (2017) 26:1296–303. 10.1044/2017_AJSLP-17-0040 29098271

[35] Ottiger B Van Wegen E Keller K Nef T Nyffeler T Kwakkel G Vanbellingen T. Getting into a “Flow” state: a systematic review of flow experience in neurological diseases. J NeuroEnginee Rehabil. (2021) 18:65. 10.1186/s12984-021-00864-w 33879182 PMC8059246

[36] Limpert E Stahel WA Abbt M. Log-normal distributions across the sciences: keys and clues: on the charms of statistics, and how mechanical models resembling gambling machines offer a link to a handy way to characterize log-normal distributions, which can provide deeper insight into variability and probability—normal or log-normal: that is the question. BioScience. (2001) 51:341–52. 10.1641/0006-3568(2001)0510341:LNDATS2.0.CO;2

[37] Buzsáki G Mizuseki K. The log-dynamic brain: how skewed distributions affect network operations. Nat Rev Neurosci. (2014) 15:264–78. 10.1038/nrn3687 24569488 PMC4051294

[38] Gualandi S Toscani G. Human behavior and lognormal distribution. A kinetic description. Math Models Methods Appl Sci. (2019) 29:717–53. 10.1142/S0218202519400049

[39] Engel GL. The clinical application of the biopsychosocial model. Am J Psychiatry. (1980) 137:535–44. 10.1176/ajp.137.5.535 7369396

[40] Danks KA Pohlig RT Roos M Wright TR Reisman DS. The relationship between walking capacity, biopsychosocial factors, self-efficacy and walking activity in individuals post-stroke. J Neurol Phys Ther. (2016) 40:232–8. 10.1097/NPT.0000000000000143 27548750 PMC5025374

[41] Kobylańska M Kowalska J Neustein J Mazurek J Wójcik B Bełza M. The role of biopsychosocial factors in the rehabilitation process of individuals with a stroke. Work. (2022) 61:523–35. 10.3233/WOR-162823 30475778 PMC6398539

[42] McNaughton H Weatherall M McPherson K Fu V Taylor WJ McRae A. The effect of the take charge intervention on mood, motivation, activation and risk factor management: analysis of secondary data from the taking charge after stroke (TaCAS) trial. Clin Rehabil. (2021) 35:1021–31. 10.1177/0269215521993648 33586474

[43] Picorelli AMA Pereira LSM Pereira DS Felício D Sherrington C. Adherence to exercise programs for older people is influenced by program characteristics and personal factors: a systematic review. J Physiotherapy. (2014) 60:151–6. 10.1016/j.jphys.2014.06.012 25092418

[44] Fors U Kamwesiga JT Eriksson GM von Koch L Guidetti S. User evaluation of a novel SMS-based reminder system for supporting post-stroke rehabilitation. BMC Med Inform Deci Mak. (2019) 19:122. 10.1186/s12911-019-0847-3 31269946 PMC6610841

[45] Swanson VA Chan V Cruz-Coble B Alcantara CM Scott D Jones M. A pilot study of a sensor enhanced activity management system for promoting home rehabilitation exercise performed during the COVID-19 pandemic: therapist experience, reimbursement, and recommendations for implementation. Int J Environ Res Public Health. (2021) 18:10186. 10.3390/ijerph181910186 34639494 PMC8508164

[46] Lobo EH Abdelrazek M Grundy J Kensing F Livingston PM Rasmussen LJ. Engagement in stroke care: opportunities and challenges in Australia and Denmark. Front Public Health. (2021) 9:758808. 10.3389/fpubh.2021.758808 34900907 PMC8661098

[47] Bailey RR. Goal setting and action planning for health behavior change. Am J Lifestyle Med. (2017) 13:615–8. 10.1177/1559827617729634 31662729 PMC6796229

[48] Magwood GS Nichols M Jenkins C Logan A Qunango S Zigbuo-Wenzler E Ellis C. Community-based interventions for stroke provided by nurses and community health workers: a review of the literature. J Neurosci Nurs. (2020) 52:152–9. 10.1097/JNN.0000000000000512 32341258 PMC7337158

[49] Lohse Keith R Lang Catherine E Boyd Lara A. Is more better? using metadata to explore dose–response relationships in stroke rehabilitation. Stroke. (2014) 45:2053–8. 10.1161/STROKEAHA.114.004695 24867924 PMC4071164

[50] Winstein C Kim B Kim S Martinez CN Schweighofer. Dosage matters. Stroke. (2019) 50:1831–7. 10.1161/STROKEAHA.118.023603 31164067 PMC12718071

[51] Jones M Collier G Reinkensmeyer DJ DeRuyter F Dzivak J Zondervan DJ Morris. Big data analytics and sensor-enhanced activity management to improve effectiveness and efficiency of outpatient medical rehabilitation, international. J Environ Res Public Health. (2020) 17:748. 10.3390/ijerph17030748 31991582 PMC7037379

