# Supplementary figures and images for: Using Large-Scale Sensor Data to Test Factors Predictive of Perseverance in Home Movement Rehabilitation: Optimal Challenge and Steady Engagement
Source: Front Neurol. 2022 Jun 20;13:896298. doi: 10.3389/fneur.2022.896298 (PMC9252527; doi:10.3389/fneur.2022.896298)

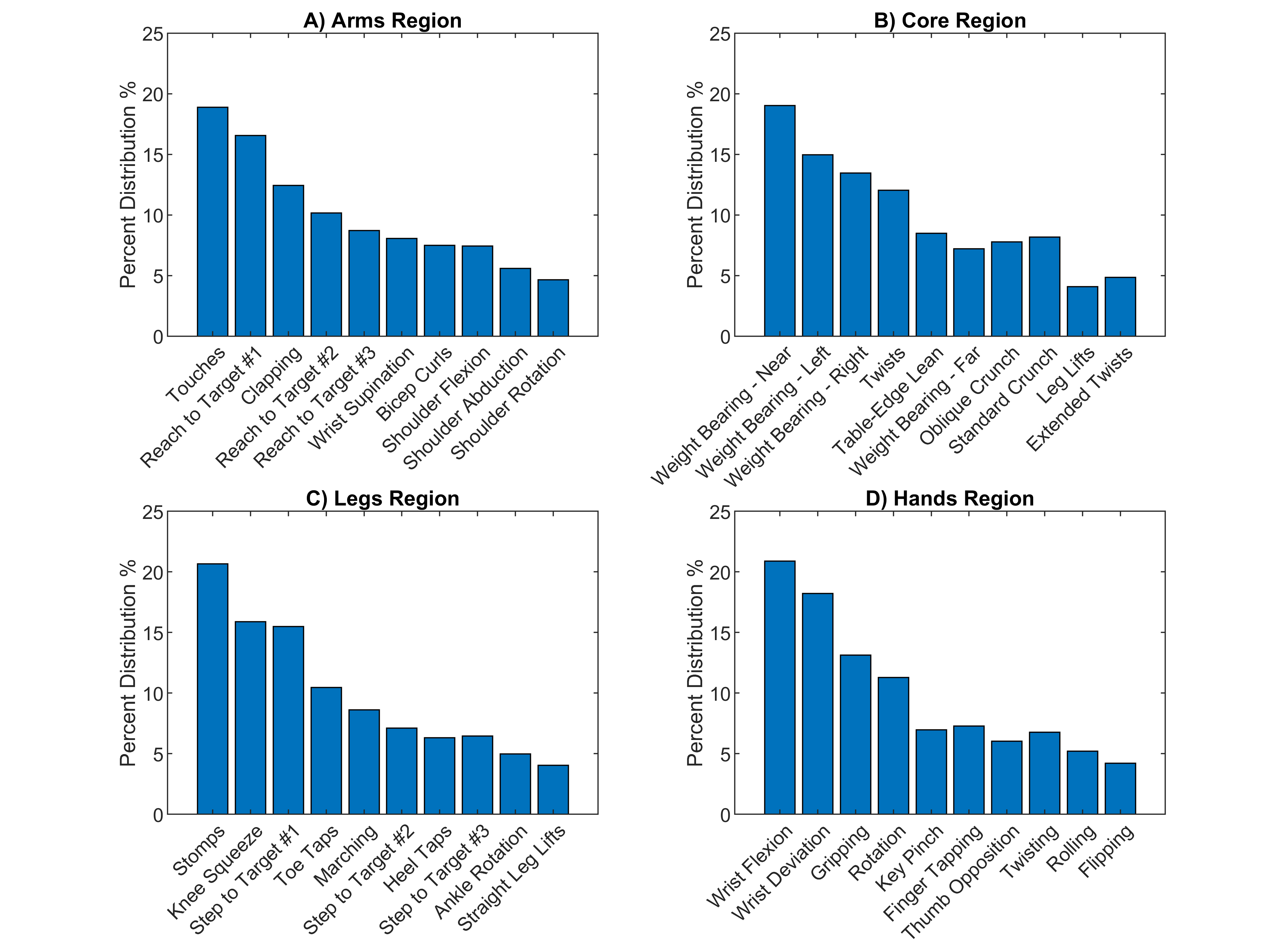

Supplement: Supplementary Figure S1 — Percent distribution of all exercises done in the (A) Arms, (B) Core, (C) Legs, and (D) Hands regions by users in the study. The exercises for each region are plotted in order of difficulty from left to right. Initially the first three exercises of each region are unlocked. As the user levels up, exercises are unlocked in order of increasing difficulty. [file Image_1.TIFF]
